# Intelligent Healthcare System Using Patients Confidential Data Communication in Electrocardiogram Signals

**DOI:** 10.3389/fnagi.2022.870844

**Published:** 2022-04-20

**Authors:** Ming Zhao, Shuo-Tsung Chen, Tzu-Li Chen, Shu-Yi Tu, Cheng-Ta Yeh, Fang-Yu Lin, Hao-Chun Lu

**Affiliations:** ^1^School of Computer Science, Yangtze University, Jingzhou, China; ^2^Department of Industrial Engineering and Management, National Taipei University of Technology, Taipei, Taiwan; ^3^Department of Mathematics, University of Michigan-Flint, Flint, MI, United States; ^4^Department of Information Management, Fu Jen Catholic University, New Taipei City, Taiwan; ^5^Graduate Institute of Business Administration, Fu Jen Catholic University, New Taipei City, Taiwan; ^6^Department of Cardiology, Linkou Chang Gung Memorial Hospital, Taoyuan, Taiwan; ^7^Department of Industrial and Business Management, Chang Gung University, Taoyuan, Taiwan

**Keywords:** multiple-coefficient, transform domain, non-linear model, simulated annealing, dementia

## Abstract

With the advent of the aging era, healthcare and elderly care have become the focus of medical care, especially the care of the elderly with dementia. Patients’ confidential data hiding is a useful technology for healthcare and patient information privacy. In this study, we implement an intelligent healthcare system using the multiple-coefficient quantization technology in transform domain to hide patients’ confidential data into electrocardiogram (ECG) signals obtained by ECG sensor module. In embedding patients’ confidential data, we first consider a non-linear model for optimizing the quality of the embedded ECG signals. Next, we apply simulated annealing (SA) to solve the non-linear model so as to have good signal-to-noise ratio (SNR), root mean square error (RMSE), and relative RMSE (rRMSE). Accordingly, the distortion of the PQRST complexes and the ECG amplitude is very small so that the embedded confidential data can satisfy the requirements of physiological diagnostics. In end devices, one can receive the ECG signals with the embedded confidential data and without the original ECG signals. Experimental results confirm the effectiveness of our method, which remains high quality for each ECG signal with the embedded confidential data no matter how the quantization size Q is increased.

## Introduction

In recent years, most countries around the world are facing the advent of the age of old age. Healthcare and long-term care have become the focus of medical care, especially the care of the elderly with dementia. Electrocardiogram (ECG) represents the human heart’s electrical activity, and hence it can be used as a reference for the analysis of cardiac pathology and cardiovascular system diagnostics. So, ECG contains a very important bio-information that has to be protected and transmitted on the Internet. It is necessary to apply information hiding technology on the ECG to protect patient rights and information.

Research on the protection of ECG information by watermarking or hiding techniques is still an important issue. Kong and Feng (2001) and Engin et al. (2005) propose a simple data hiding method for ECG signals, but the method is not blind. Zheng and Qian (2008) and Zheng et al. (2009) proposed a wavelet-domain ECG data hiding method in non-QRS complex frames to ensure the restoration of almost un-distorted ECG signals. Kaur et al. (2010) presented the safe transmission of ECG signals in wireless networks by using a blind hiding method. Ibaida et al. (2010) improved the watermarking technique of least significant bit (LSB) and applied this improved technique to hide healthcare information in an ECG signal. Ibaida et al. (2011) presented a watermarking technique to embed patient biomedical information into ECG signals so as to ensure patient/ECG linkage integrity, and is suitable for a wearable sensor-net health monitoring system. However, the selection of embedding location is difficult.

In Guo and Zhou (2012) and He et al. (2012), a Haar wavelet transform with 7 levels decomposition is adopted to transform the ECG signal, and then the synchronization code combined with watermark are embedded into the low-frequency sub-band of level 7 to have good signal-to-noise ratio (SNR) and bit error rate (BER). However, the quality of all watermarked ECG signals decreases when the embedding strength increases. Moreover, Guo and Zhou (2012) presented a model of single-channel electromyography blind recognition. Dey et al. (2012a) embedded reversible binary bits to be watermark in the photoplethysmography (PPG) signal and extracted them by an error prediction algorithm. Dey et al. (2012b) presented a new session-based blind watermarking scheme by hiding a binary watermark image into the ECG signal. However, the methods in Dey et al. (2012a,b) are not blind. Ayman and Ibrahim (2013) developed a wavelet-based information hiding technique to protect patient confidential data by combining encryption and scrambling. Their method applied wavelet transform to ECG signal to hide the related patient confidential data and physiological information. In Chen et al. (2014) and Tseng et al. (2014), single-coefficient quantization in transform domain is applied to the digital watermark encryption technology on the ECG for protecting patient rights and information. By this method, the changes in the PQRST complexes and amplitude of the ECG signal are very small. Jero et al. (2015) and Jero and Ramu (2016) used curvelet transforms to identify the coefficients that store the crucial information about diagnosis. The novelty of their approach is the usage of curvelet transform for ECG steganography, adaptive selection of watermark location, and a new threshold selection algorithm. In Swierkosz and Augustyniak (2018), an original time-frequency watermarking technique with an adaptive beat-to-beat lead-independent data container design is implemented. The authors tested six wavelets, six coding bit depth values, and two types of watermark content to find the conditions that are necessary for watermarked ECG to maintain the compliance with International Electrotechnical Commission (IEC) requirements for interpretation performance. Sanivarapu et al. (2020) proposed a wavelet method-based watermarking scheme for patient information hiding in the ECG as a QR image. They first converted the 1D-ECG signal to 2D-ECG image using Pan–Tompkins algorithm, and then used a wavelet transform to decompose the 2D-ECG image. They then decompose the detail coefficient of wavelet and the QR image using QR decomposition for embedding data.

In this study, we install ECG sensor module on patients to obtain their ECG and propose a new information hiding technology for the ECG. As ECG has high requirements for accuracy, we rewrite SNR and amplitude-quantization as performance index and constraint to obtain an optimization model for embedding patients’ confidential data into ECGs imperceptibly. The optimization model is then solved by simulated annealing (SA) algorithms and applied to embed patients’ confidential data. By network transmission, one can receive the ECGs embedded with the confidential data in the other end and extract the confidential data without the original ECG. In experiments, we evaluated the relation between embedding strength Q and SNR, embedding strength Q and root mean square error (RMSE), and embedding strength Q and similarity. Experimental results confirm the effectiveness of our method, which remains high quality for each ECG signal with the embedded confidential data no matter how the quantization size Q is increased.

The rest of this article is as follows. Section 2 reviews some preliminaries including ECG principle, discrete wavelet transform (DWT), discrete Fourier transform (DFT), discrete cosine transform (DCT), and SA. Section 3 presents the proposed SA-based quantization embedding method for hiding patient confidential data into ECG signals. Section 4 shows experimental results and discussion. Conclusions are finally drawn in Section 5.

## Preliminaries

In this section, we review some preliminaries including ECG principle, DWT, DFT, DCT, and SA.

### Electrocardiogram Signal

**FIGURE 1:**
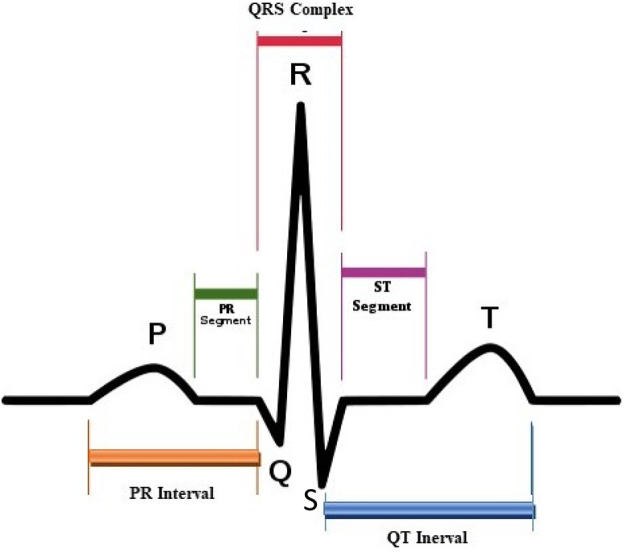
ECG signal.

The abbreviation ECG denotes the electrocardiogram wave, named by the Dutch physiologist W. Einthoven (the inventor of the ECG). As shown in Figure 1, he classified one cardiac cycle into P, Q, R, S, and T complex waves (Burrus et al., 1998). The ECG diagnosis mainly depends on the PQRST waves. Therefore, it is necessary to maintain the shape of these waveforms when we add information into ECG signals or perform ECG signals compression.

### Discrete Wavelet Transform

The DWT is a technique attained by scales and translates a mother wavelet ψ(*x*). The normalized scaling functions and wavelets are defined as,


$$\varphi_{i,n}\,\left(t\right)=2^{i\,/\,2}\,h_{i}\,\varphi \,\left(2^{i}\,\text{t-n}\right)$$

$$\psi_{i,n}\,\left(t\right)=2^{i\,/\,2}\,g_{i}\,\psi \,\left(2^{i}\,\text{t-n}\right)$$

where *i* and *n* denote the scale and translation parameters, and *h*_*i*_ and *g*_*i*_are the low-pass and high-pass filters, respectively. It should be noted that orthogonal wavelet basis functions are adopted to ease the work in calculating coefficients expansion so that an input signal can be decomposed into several non-overlapping multi-resolution sub-bands, including low-frequency sub-bands and high-frequency sub-bands (Oran Brigham, 1988; Mallat, 1989, 1999).

### Discrete Fourier Transform

The DFT converts a finite sequence of equally spaced samples of a function into a same-length sequence of equally spaced samples of the discrete-time Fourier transform (DTFT), which is a complex-valued function of frequency. Since it deals with a finite amount of data, it can be implemented in computers by numerical algorithms or even dedicated hardware. These implementations usually employ efficient fast Fourier transform (FFT) algorithms, so much so that the terms “FFT” and “DFT” are often used interchangeably (Rao and Yip, 1990; Oppenheim et al., 1999).

### Discrete Cosine Transform

The DCT expresses a finite sequence of data points in terms of a sum of cosine functions oscillating at different frequencies. In particular, a DCT is a Fourier-related transform similar to the DFT but using only real numbers (Rao and Yip, 1990; Granville et al., 1994).

### Simulated Annealing

Simulated annealing is an artificial intelligent algorithm, which utilizes probabilistic concept to approximate the global optimum of a given function. Specifically, It uses a metaheuristic method to approximate global optimization in a large discrete search space. In general, SA algorithm works as follows. At each time step, SA algorithm gives a solution randomly close to the current one, evaluates its quality, and then decides to move to it or to stay with the current solution based on either one of two probabilities between which it chooses based on the fact that the new solution is better or worse than the current one. During the search, the temperature is progressively decreased from an initial positive value to zero, and this affects the two probabilities: at each step, the probability of moving to a better new solution is either kept as 1 or is changed toward a positive value; instead, the probability of moving to a worse new solution is progressively changed toward zero (Bouttier and Gavra, 2019). The implementation of SA algorithm is easy because of its simple concept and computation.

## Proposed Intelligent Healthcare System

**FIGURE 2:**
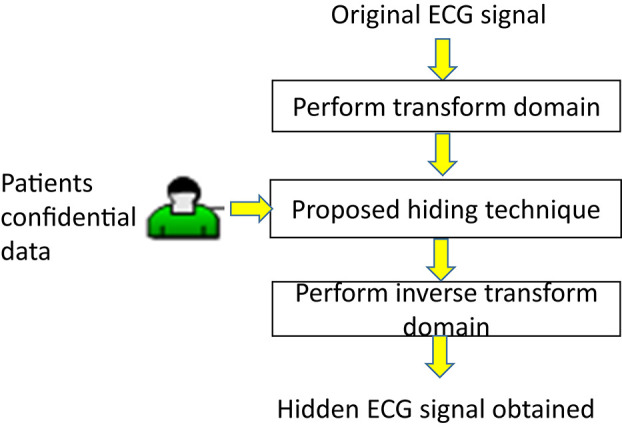
A flowchart of the proposed method.

This section first shows the proposed amplitude-quantization hiding technique for confidential data communication and private information protection in the transform domain of ECGs, as shown in Figure 2. Next, we provide the architecture of the proposed intelligent multiple-coefficient quantization hiding system for patients’ confidential data communication.

### Information Hiding

As shown in Figure 2, the information hiding and detection proposed in this study is introduced as follows. Let *S* = {*s*_1_,*s*_2_,⋯,*s*_*N*_} denote an ECG signal with total length *N* sample points. We then perform three transforms, DWT, DCT, and DFT, independently on the ECG signal*S* = {*s*_1_,*s*_2_,⋯,*s*_*N*_}so that the binary bits *B* = {*m*_*i*_} can be hidden into the coefficients of each transform domain. Finally, the hidden ECG signal is obtained and denoted by $\tilde{S}=\left\{{\tilde{s}}_{1},{\tilde{s}}_{2},\cdots ,{\tilde{s}}_{N}\right\}$. The details of the hiding process for each transform are listed below.

*DWT*: In the information hiding, we first apply Haar DWT with orthogonal wavelet bases to decompose an ECG signal into several non-overlapping sub-bands. In order not to distort or disturb the original ECG as much as possible, we hide the binary bits *B* = {*m*_*i*_} into the DWT lowest-frequency coefficients by the following rule,


$$\sum_{i=1}^{N}\left|{\tilde{c}}_{i}\right|=\left\{ \begin{array}{c} \left\lfloor \sum_{i=1}^{N}\left|c_{i}\right|\,/\,Q\right\rfloor \,Q+3\,Q\,/\,4,\mathrm{if}\,m_{i}=1\\ \left\lfloor \sum_{i=1}^{N}\left|c_{i}\right|\,/\,Q\right\rfloor \,Q+Q\,/\,4,\mathrm{if}\,m_{i}=0 \end{array}\right.$$

where {*c*_*i*_} and $\left\{{\tilde{c}}_{i}\right\}$ are the DWT lowest-frequency coefficients before and after hiding, respectively; *Q* is the hiding strength. By performing the inverse DWT (IDWT), the hidden ECG signal $\tilde{S}$ is obtained and the information hiding is completed.

*DCT:* The hiding rule for DCT multiple coefficients is the same as for DWT. Similarly, we hide the binary bits *B* = {*m*_*i*_} into the DCT lowest-frequency coefficients {*c*_*i*_}, respectively.

*DFT:* The hiding rule for DFT multiple coefficients is the same as for DWT. Similarly, we hide the binary bits *B* = {*m*_*i*_} into the DFT lowest-frequency coefficients {*c*_*i*_}, respectively.

Theoretically, an ECG signal is modified when embedding patient confidential information. The modification is usually called a distortion. In order to reduce the distortion of ECG signal, we consider the maximum of SNR which is defined as,


$$\begin{array}{c} S\,N\,R=-10\,\log\left[\sum_{i=1}^{N}{\left({\tilde{s}}_{i}-s_{i}\right)}^{2}\,/\,\sum_{i=1}^{N}s_{i}^{2}\right] \end{array}$$

where {*s*_*i*_} represents the original ECG signal sample points, and $\left\{{\tilde{s}}_{i}\right\}$ represents the unknown embedded (or modified) ECG signal sample points. Since we implement the DWT with orthogonal wavelet bases, the SNR can be rewritten to the form,


$$\begin{array}{c} S\,N\,R=-10\,\log\left[\sum_{i=1}^{N}{\left(\left|{\tilde{c}}_{i}\right|-\left|c_{i}\right|\right)}^{2}\,/\,\sum_{i=1}^{N}{\left|c_{i}\right|}^{2}\right] \end{array}$$

From the point of view on maximizing SNR, we consider to determine the unknown values of ${\left\{{\tilde{c}}_{i}\right\}}_{i=0}^{N}$ by using the following optimization models.

• If the bit *b*_*i*_ = 1 is embedded into {*c*_*i*_|1≤*i*≤*N*}, $\sum_{i=1}^{N}\left|c_{i}\right|$ is modified to $\sum_{i=1}^{N}\left|{\tilde{c}}_{i}\right|$ by, maximize $\begin{array}{c} -10\,\log\left[\sum_{i=1}^{N}{\left(\left|{\tilde{c}}_{i}\right|-\left|c_{i}\right|\right)}^{2}\,/\,\sum_{i=1}^{N}{\left|c_{i}\right|}^{2}\right] \end{array}$ subject to $\sum_{i=1}^{N}\left|{\tilde{c}}_{i}\right|=\left\lfloor \sum_{i=1}^{N}\left|c_{i}\right|\,/\,Q\right\rfloor \,Q+\frac{3}{4}\,Q\,$
• If the bit *b*_*i*_ = 1 is embedded into {*c*_*i*_|1≤*i*≤*N*}, $\sum_{i=1}^{N}\left|c_{i}\right|$ is modified to $\sum_{i=1}^{N}\left|{\tilde{c}}_{i}\right|$ by, maximize $\begin{array}{c} -10\,\log\left[\sum_{i=1}^{N}{\left(\left|{\tilde{c}}_{i}\right|-\left|c_{i}\right|\right)}^{2}\,/\,\sum_{i=1}^{N}{\left|c_{i}\right|}^{2}\right] \end{array}$ subject to $\sum_{i=1}^{N}\left|{\tilde{c}}_{i}\right|=\left\lfloor \sum_{i=1}^{N}\left|c_{i}\right|\,/\,Q\right\rfloor \,Q+\frac{1}{4}\,Q\,$

Due to the fact that the algorithm of SA is easy to be implemented by its simple concept and calculation mentioned in section “Simulated Annealing,” especially for embedded system, we adopt SA to solve the optimization models approximately. In other words, we apply SA to obtain the optimal solutions of ${\left\{{\tilde{c}}_{i}\right\}}_{i=0}^{N}$ approximately. At each time step in SA, the algorithm randomly selects a solution close to the current one, measures its quality, and then decides to move to it or to stay with the current solution based on either one of two probabilities between which it chooses on the basis of the fact that the new solution is better or worse than the current one. During the search, the temperature is progressively decreased from an initial positive value to zero, which affects the two probabilities: at each step, the probability of moving to a better new solution is either kept as 1 or is changed toward a positive value; instead, the probability of moving to a worse new solution is progressively changed toward zero. In this section, we adopt SA to approach the optimal solution of the proposed optimization model. The detailed procedure of SA in solving the proposed optimization model is introduced in the following steps.

Step 1: Setting the initial value for parameters including initial solution *C*_0_, initial temperature *T*_0_, final temperature *T*_*f*_, cooling rate *r*, and number of iteration *D* for each temperature *T*, where *T*_0_≤*T*≤*T*_*f*_.
Step 2: For each *d = 1,…,D* in a temperature *T*, do the following repeatedly:
  (1) Produce a new solution $C^{\prime \prime }=\left\{\left|c_{1}^{\prime \prime }\right|,\left|c_{2}^{\prime \prime }\right|,\cdots ,\left|c_{n}^{\prime \prime }\right|\right\}$ randomly and calculate the difference $\Delta E=E\left(C^{\prime \prime }\right)-E\left(C^{\prime }\right)=\{-10\log\left[\sum_{i=1}^{n}{\left(\left|c_{i}^{\prime \prime }\right|-\left|c_{i}\right|\right)}^{2}/\sum_{i=1}^{N}{\left|c_{i}\right|}^{2}\right]-$$\left(-10\log\left[\sum_{i=1}^{N}{\left(\left|c_{i}^{\prime }\right|-\left|c_{i}\right|\right)}^{2}/\sum_{i=1}^{N}{\left|c_{i}\right|}^{2}\right]\right)\}$ between current solution $C^{\prime }=\left\{\left|c_{1}^{\prime }\right|,\left|c_{2}^{\prime }\right|,\cdots ,\left|c_{n}^{\prime }\right|\right\}$ and the new solution (neighbors)$C^{\prime \prime }=\left\{\left|c_{1}^{\prime \prime }\right|,\left|c_{2}^{\prime \prime }\right|,\cdots ,\left|c_{n}^{\prime \prime }\right|\right\}$.
  (2) The probability of making the transition from the current solution to a new solution (neighbors) is specified by an acceptance probability function *P*(Δ*E*,*T*) that depends on Δ*E* and *T*.

$$P\,\left(\Delta \,E,T\right)=\left\{ \begin{array}{cc} 1, & i\,f\,\,\Delta \,E\leq 0\\ e^{\left(\frac{-\Delta \,E}{T}\right)}, & i\,f\,\,\Delta \,E>0 \end{array}\right.$$

In case Δ*E*≤0, the probability function *P*(Δ*E*,*T*) is equal to 1 indicating that the current solution *S* is replaced by new solution *S’*. In case Δ*E* > 0, the current solution *S* is replaced by the new solution *S’* when probability function $P\,\left(\Delta \,E,T\right)=e^{\left(\frac{-\Delta \,E}{T}\right)}$ is greater than a threshold *H*∈(0,1).

Step 3: When step 2 is finished, the temperature *T* is decreased by a cooling rate *r* to a new temperature *T = rT*.
Step 4: Check if temperature *T* reaches the final temperature *T*_*f*_ to stop SA.

After applying SA to embed patients’ essential body functions into ECG signal *S*, we obtain an embedded ECG signal $\tilde{S}$, respectively.

### Information Detection

In the information detection, similar to the information hiding mentioned earlier, we first perform the transforms, DWT, DCT, and DFT on the test ECG signals, respectively. Next, we detect binary bits $\left\{m_{i}^{*}\right\}$ from the hidden coefficients $\left\{{\tilde{c}}_{i}\right\}$ of the DWT, DCT, and DFT by the following rule:


$$m_{i}^{*}=\left\{ \begin{array}{c} 1,\mathrm{if}\,\sum_{i=1}^{N}\left|{\tilde{c}}_{i}\right|-\left\lfloor \sum_{i=1}^{N}\left|{\tilde{c}}_{i}\right|\,/\,Q\right\rfloor \,Q\geq Q\,/\,2\\ 0,\mathrm{if}\,\sum_{i=1}^{N}\left|{\tilde{c}}_{i}\right|-\left\lfloor \sum_{i=1}^{N}\left|{\tilde{c}}_{i}\right|\,/\,Q\right\rfloor \,Q<Q\,/\,2 \end{array}\right.$$

### Architecture of the Proposed System

**FIGURE 3:**
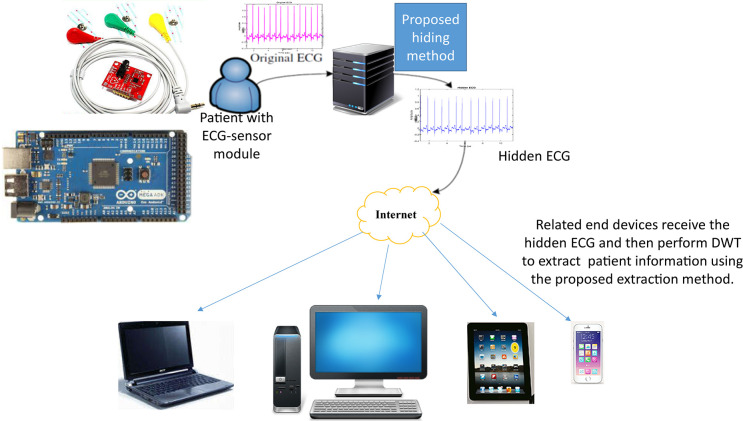
Proposed intelligent multiple-coefficient quantization hiding and communication system for patients’ confidential data.

Figure 3 shows the architecture of the proposed intelligent multiple-coefficient quantization hiding system for patients’ confidential data communication. First, we install ECG-sensor module, including electrode patch, AD8232 ECG sensor, and Arduino, on patients or dementia patients to obtain their ECG. As the ECG-sensor module shown in the upper left corner of Figure 3, three electrode patches are installed on patient body and connected to AD8232 ECG sensor. At the same time, AD8232 is connected to Arduino which outputs the ECG data to a computer. Next, as in subsections “Information Hiding” and “Information Detection,” we hide the patient’s information into the ECG signal in the transform domain by performing DWT/DFT/DCT and IDWT/inverse DFT (IDFT)/inverse DCT (IDCT). At the other end, the embedded patients confidential data are extracted after the hidden ECG is received through wireless communication and we perform transform domain DWT/DFT/DCT on the hidden ECG.

## Experiments and Discussion

In experiments, we apply the ECG data obtained from ECG sensor module to test the proposed method on each ECG signal with length 4,096 samples represented by 16-bit. To have more embedding capacity, we set *N* to 2. The evaluation of experimental results and discussion are listed in section 4.1. Limitation and future research plan are listed in section 4.2.

### Experimental Results and Discussion

Without generality, the performance of the proposed scheme is evaluated by SNR, similarity, and RMSE, which are formulated as follows:


$$\text{SNR}=-10\,\log_{10}\left(\frac{\sum_{i=1}^{N}{\left({\tilde{s}}_{i}-s_{i}\right)}^{2}}{\sum_{i=1}^{N}s_{i}^{2}}\right),$$

$$\text{Similarity}\,\left(S,\tilde{S}\right)=\frac{\sum_{i=1}^{N}s_{i}\,{\tilde{s}}_{i}}{\sum_{i=1}^{N}{\tilde{s}}_{i}^{2}},$$

$$\text{RMSE}=\sqrt{\frac{1}{N}\,\sum_{i=1}^{N}{\left({\tilde{s}}_{i}-s_{i}\right)}^{2}},$$

where *s*_*i*_ and ${\tilde{s}}_{i}$ denote original ECG signal sample point and hidden ECG signal sample point, respectively.

**FIGURE 4:**
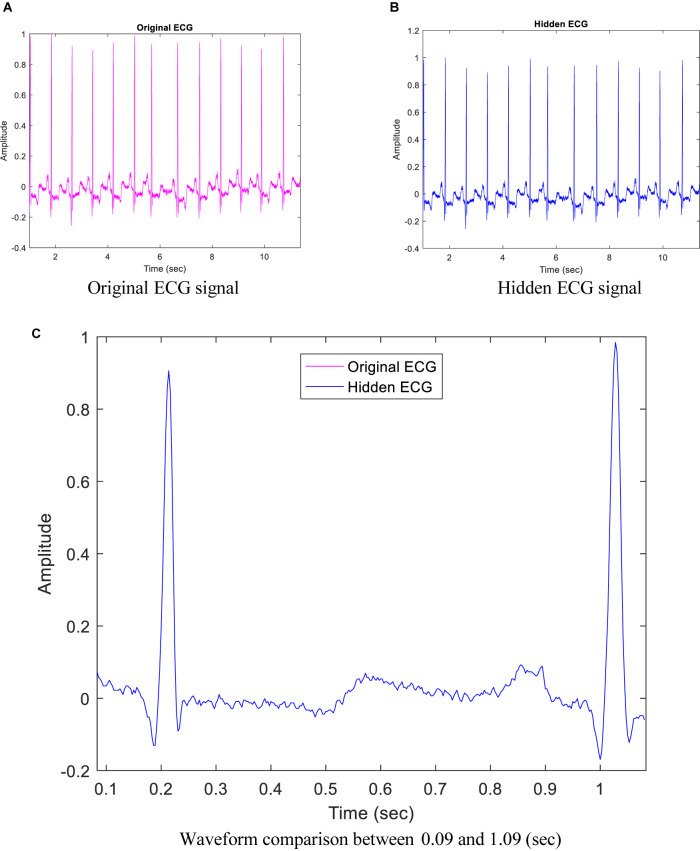
Comparison between original electrocardiogram (ECG) signal and hidden ECG signal for dataset ID 1. **(A)** Original ECG signal. **(B)** Hidden ECG signal. **(C)** Waveform comparison between 0.09 and 1.09 s.

The proposed method provides good quality for each hidden ECG signal. For example, Figures 4A,B show the original ECG signal and hidden ECG signal using DWT lowest-frequency coefficients in 5-level decomposition for dataset ID 1. They look almost indistinguishable. As shown in Figure 4C, the purple curve represents the original ECG signals, and the blue curve represents the hidden ECG signals using DWT and quantization size *Q* = 4000.

Moreover, as shown in Table 1, the proposed method applies SA to optimize the quality of each hidden ECG signal and then improve the drawback that the quality of each hidden ECG signal is greatly reduced as the quantization size *Q* is increased. In other words, our method remains high quality, with good RMSE and SNR, for each hidden ECG signal under sufficient hiding capacity no matter how the quantization size *Q* is increased. In addition, both DFT and DCT also have the same effect on RMSE and SNR.

**TABLE 1:** Experimental results for the three transforms: discrete wavelet transform (DWT), discrete cosine transform (DCT), and discrete Fourier transform (DFT).

ID	Method	Domain	Q	Amplitude similarity	SNR	RMSE	Interval RMSE in ECG			
							PR	QRS	ST	QT
1	Chen et al., 2014	DWT (Level 5)	500	1	40.26	36.86	0	0	0.028	0
			2000	1	32.74	69.64	0	0	0	0
			10000	1	20.75	126.13	0	0	0	0
		DFT	500	1	61.13	3.74	0	0	0	0
			2000	1	50.09	7.49	0	0	0	0
			10000	0.99	38.11	29.79	0	0	0	0
		DCT	500	0.99	26.62	198.90	0	0	0	0
			2000	0.98	17.85	434.41	0	0	0	0
			10000	0.81	4.73	1964.4	0	0	0	0
	**Proposed**	DWT (Level 5)	500	0.99	38.46	50.92	0	0	0	0
			2000	0.99	38.44	50.99	0	0	0	0
			10000	0.99	38.55	50.41	0	0	0	0
		DFT	500	1	33.74	91.61	0	0	0	0
			2000	1	31.73	110.41	0	0	0	0
			10000	1	32.09	106.01	0	0	0	0
		DCT	500	1	46.08	21.17	0	0	0	0
			2000	1	45.70	22.10	0	0	0	0
			10000	1	45.58	22.41	0	0	0	0
2	Chen et al., 2014	DWT (Level 5)	500	1	40.78	35.95	0	0.002	0.002	0
			2000	1	31.67	72.57	0	0	0	0
			10000	1	20.31	141.47	0	0	0	0
		DFT	500	1	60.01	3.72	0	0	0	0
			2000	1	51.56	7.35	0	0	0	0
			10000	1	38.33	30.03	0	0	0	0
		DCT	500	0.99	25.68	204.19	0	0	0	0
			2000	0.98	16.94	443.70	0	0	0	0
			10000	0.78	2.94	1982.1	0	0	0	0
	**Proposed**	DWT (Level 5)	500	1	32.59	92.49	0	0	0	0
			2000	1	32.71	91.01	0	0	0	0
			10000	0.99	32.29	95.49	0	0	0	0
		DFT	500	0.99	31.36	106.28	0	0	0	0
			2000	0.99	31.76	101.53	0	0	0	0
			10000	0.99	32.19	96.61	0	0	0	0
		DCT	500	1	35.13	68.85	0	0	0	0
			2000	1	35.33	67.26	0	0	0	0
			10000	1	35.27	67.53	0	0	0	0
3	Chen et al., 2014	DWT (Level 5)	500	0.99	43.10	34.84	0	0	0	0
			2000	0.99	35.05	69.96	0	0	0	0
			10000	0.99	20.46	265.70	0	0	0	0
		DFT	500	1	62.52	3.72	0	0	0	0
			2000	1	51.49	7.45	0	0	0	0
			10000	1	39.42	29.95	0	0	0	0
		DCT	500	0.99	28.02	197.89	0	0	0	0
			2000	0.99	19.42	423.11	0	0	0	0
			10000	0.87	5.43	1886.2	0	0	0	0
	**Proposed**	DWT (Level 5)	500	1	34.25	96.58	0	0	0	0
			2000	1	34.23	96.84	0	0	0	0
			10000	1	32.83	113.84	0	0	0	0
		DFT	500	0.99	27.32	302.95	0	0	0	0
			2000	0.99	26.54	331.65	0	0	0	0
			10000	0.99	27.61	293.19	0	0	0	0
		DCT	500	1	28.27	169.43	0	0	0	0
			2000	1	28.36	167.42	0	0	0	0
			10000	1	28.54	166.77	0	0	0	0
5	Chen et al., 2014	DWT (Level 5)	500	1	40.56	36.07	0.002	0.002	0	0
			2000	1	33.65	63.48	0	0	0	0
			10000	1	19.05	270.75	0	0	0	0
		DFT	500	1	62.23	3.74	0	0	0	0
			2000	1	52.16	7.53	0	0	0	0
			10000	0.99	38.22	29.77	0	0	0	0
		DCT	500	0.99	27.27	209.75	0	0	0	0
			2000	0.99	19.62	450.70	0	0	0	0
			10000	0.85	3.76	1980.2	0	0	0	0
	**Proposed**	DWT (Level 5)	500	1	26.21	258.11	0	0	0	0
			2000	1	26.95	238.72	0	0	0	0
			10000	1	26.78	253.62	0	0	0	0
		DFT	500	0.99	28.32	156.43	0	0	0	0
			2000	0.99	28.54	157.12	0	0	0	0
			10000	0.99	28.61	156.37	0	0	0	0
		DCT	500	1	34.17	94.74	0	0	0	0
			2000	1	34.11	95.42	0	0	0	0
			10000	1	34.25	94.08	0	0	0	0
6	Chen et al., 2014	DWT (Level 5)	500	0.99	41.56	37.21	0.051	0	0	0
			2000	0.99	34.20	68.96	0	0	0	0
			10000	0.99	20.67	260.17	0	0	0	0
		DFT	500	1	62.46	3.76	0.697	0.019	0.019	0
			2000	1	53.47	7.50	0	0	0	0
			10000	0.99	40.45	29.62	0	0	0	0
		DCT	500	0.99	27.84	202.645	0	0	0	0
			2000	0.99	19.26	431.98	0	0	0	0
			10000	0.86	3.41	1897.0	0	0	0	0
	**Proposed**	DWT (Level 5)	500	0.99	26.16	317.65	0	0	0	0
			2000	0.99	26.34	327.15	0	0	0	0
			10000	0.99	26.76	322.48	0	0	0	0
		DFT	500	0.99	27.18	166.33	0	0	0	0
			2000	0.99	27.09	167.05	0	0	0	0
			10000	0.99	27.46	166.46	0	0	0	0
		DCT	500	1	31.41	134.54	0	0	0	0
			2000	1	32.26	129.75	0	0	0	0
			10000	1	32.18	128.96	0	0	0	0
7	Chen et al., 2014	DWT (Level 5)	500	1	41.96	33.04	0	0	0	0
			2000	1	35.15	68.28	0	0	0	0
			10000	0.99	20.75	284.63	0	0	0	0
		DFT	500	1	64.46	3.70	0	0	0	0
			2000	1	55.37	7.47	0	0	0	0
			10000	1	41.24	30.21	0	0	0	0
		DCT	500	0.98	29.58	205.58	0	0	0	0
			2000	0.99	19.76	450.82	0	0	0	0
			10000	0.88	5.43	2092.6	0	0	0	0
	**Proposed**	DWT (Level 5)	500	1	28.83	283.36	0	0	0	0
			2000	1	28.39	297.09	0	0	0	0
			10000	1	28.56	291.09	0	0	0	0
		DFT	500	0.99	25.96	160.33	0	0	0	0
			2000	0.99	25.74	161.05	0	0	0	0
			10000	0.99	25.36	161.46	0	0	0	0
		DCT	500	1	46.58	29.02	0	0	0	0
			2000	1	46.00	31.05	0	0	0	0
			10000	1	46.22	28.54	0	0	0	0

### Limitation and Future Research Plan

There are some limitations in the proposed system. The two limitations in our system are that the ECG signals embedded with patients’ confidential data are transmitted to the receiver with an upload speed with a limit of 75 Mbps and a download speed with a limit of 300 Mbps. In future research plan, we will reduce the size of the sensor module and the computer and have a waterproof case without affecting the function.

## Conclusion

Based on the proposed optimization model and SA algorithm, we apply multiple-coefficients quantization technology to propose a new method for embedding patients’ confidential data into ECG signals in the transform domain. After testing ECG data sets by using the proposed embedding method, the difference between the embedded ECG signal and the original one is very small and negligible for physiological diagnostics. In addition, the proposed method improves the drawback that the quality of each embedded ECG signal is greatly reduced as the quantization size Q is increased. At the end devices, the embedded patients’ confidential data are extracted after the embedded ECG is received through internet transmission and we perform DWT on the embedded ECG.

## Data Availability Statement

The original contributions presented in the study are included in the article/supplementary material, further inquiries can be directed to the corresponding author.

## Author Contributions

S-TC: conceptualization and methodology. S-TC, F-YL, and T-LC: software. S-TC, C-TY, and S-YT: validation. S-TC, MZ, and H-CL: writing – review and editing. All authors have read and agreed to the published version of the manuscript.

## Conflict of Interest

The authors declare that the research was conducted in the absence of any commercial or financial relationships that could be construed as a potential conflict of interest.

## Publisher’s Note

All claims expressed in this article are solely those of the authors and do not necessarily represent those of their affiliated organizations, or those of the publisher, the editors and the reviewers. Any product that may be evaluated in this article, or claim that may be made by its manufacturer, is not guaranteed or endorsed by the publisher.
